# Comment on “System Accuracy Evaluation of the GlucoRx Nexus Voice TD-4280 Blood Glucose Monitoring System”

**DOI:** 10.1155/2015/538785

**Published:** 2015-03-17

**Authors:** Nilesh Nathwani

**Affiliations:** GlucoRx Ltd., 4 Wintonlea, Monument Way West, Woking, Surrey GU21 5EN, UK

The Khan et al. study [1] uses an inappropriately designed and uncontrolled evaluation protocol which cannot be used to demonstrate a blood glucose meter's accuracy and draw valid conclusions regarding compliance with ISO 15197:2013 standards.

Documents that list best practice quality guidelines or recommendations for undertaking and reporting appropriate evaluations of blood glucose system accuracy are available from several sources [2–4]. Limitations in study design and protocols can lead to differences in results inappropriately being attributed to blood glucose system inaccuracy. In common with many other evaluation studies, Khan et al. do not address many of the variables that can adversely impact quality and validity of conclusions.

Evaluation of accuracy and performance of blood glucose systems is complex, and testing must be carefully designed and performed [5]. Performing studies correctly in accordance with guidelines assumes greater importance as accuracy demands increase and tighter standards are introduced. A wide range of variables must be taken into account to ensure that any inaccuracy of results is due to the blood glucose system and not due to other factors such as the reference method, variations in the specimens compared, or experimental artefact. Performing a thorough reference method evaluation, testing blood glucose meter system and reference samples in duplicate, and control of elapsed time and glycolysis are important [2].

Independent appropriate evaluation of the accuracy of blood glucose meters is invaluable to healthcare professionals and manufacturers. They provide independent verification of accuracy claims at system launch, assess the performance of different lots of strips produced, and allow comparison of different systems. The use of carefully controlled detailed protocols is however essential to allow appropriate assessment of accuracy claims against the ISO 15197:2013 standard. Correct evaluation is complex and each publication must be examined in detail to establish any variation from recommended study designs and to establish their quality and the validity of conclusions drawn. Thorpe (2013) [5] provides an overview of frequently made evaluation protocol errors and easy-to-use checklists to verify the quality of blood glucose meter evaluation studies. Use of the accuracy checklist proposed by Thorpe clearly indicates that the evaluation protocol used by Khan et al. does not allow correct assessment of accuracy against ISO 15197:2013 to which manufacturers are required to adhere in order to demonstrate the accuracy and performance of commercially available systems. The use of an inappropriate study design results in apparent contradiction of results with the system's claims and creates confusion. The authors acknowledge that protocol limitations including the measurement error inherently attributable to the comparative method used may potentially reduce the reliability of their results.

Meter results should be compared against results generated by the reference method specified by the manufacturers [5, 6]. Five percent differences are common if inappropriate reference methods are used, and laboratory comparison methods for blood glucose can have a total error of up to 10% [7]. Khan et al. compare blood glucose results from the meter system against those from the UniCel DxC 800 clinical system. This, however, is a laboratory comparison method and is neither a true reference method nor the manufacturer's standing measurement procedure which for the GlucoRx system is the YSI analyser. 3–8% differences are reported if inappropriate comparison methods are used [5]. No indication of the comparison method's analytical performance, bias, imprecision, total error [8], or its traceability to higher reference methods is provided. The trueness of the reference method should be checked with National Institute of Standards and Technology reference materials or other traceable materials. Reference sample analysis should be performed in at least duplicate and checked for differences [5, 6]. No indication is given by Khan et al. over what time period samples were analysed on the laboratory comparison assay and the number of runs or batches involved.

The paper incorrectly states that the GlucoRx system is based on the enzyme glucose oxidase whereas it is in fact based on glucose dehydrogenase.

Although the evaluation compares “like with like” samples, it uses venous blood rather than fresh capillary whole blood samples as specified for accuracy studies in the ISO standard [9]. Any anticoagulant used is not specified and samples are not assayed in duplicate. Significantly, no indication is given of time delays between sample collection and analysis on the glucose meter and analysis on the laboratory based comparison method. The paper states that samples were “then sent for determination of plasma glucose using the reference method.” Guidelines clearly state that the reference samples should be tested within an adequate time [4] of the meter tests, centrifuged within 5 min of the meter tests and the plasma removed, or stabilised (e.g., by a validated deproteinisation method) for later measurements [5]. It is important that there should be minimal delay in analysis and postcollection control of sample handling time because glycolysis in whole blood samples can cause rapid glycaemic change dependent on the haematocrit [10]. If delays are not controlled, differences in the data can be due to glucose concentration differences in the comparative samples instead of differences between the two methods [5].

Although the Khan et al. protocol uses samples from over 100 patients, it does not assay samples in duplicate on the glucose meter and therefore does not provide the 200 data points required for each lot of strips examined. ISO 15197:2013 [9] also specifies that 3 different lots of strips should be used to demonstrate minimum accuracy performance and hence requires 600 data points. ISO 15197 importantly specifies an appropriate distribution of sample results, spanning the analytical range with appropriate percentages of results within specific concentration intervals, which should be analysed. The Khan et al. study fails to demonstrate an appropriate spread of results from patient samples with sufficient numbers of samples at low, medium, and high glucose concentrations and above/below the threshold level.

In conclusion, the significant differences in protocol between the Khan et al. study and that specified by ISO 15197 as needed to determine accuracy clearly invalidate the study conclusions that the GlucoRx Nexus Voice system does not meet the minimum accuracy requirements specified for CE marking. Khan et al. claim that their study demonstrates the importance of internally validating the accuracy of a blood glucose meter. Whilst appropriate independent evaluation of accuracy is essential, such evaluations are complex. The Khan et al. study clearly shows how use of an inappropriate study design and protocol can produce invalid, inappropriate conclusions. A small number of correctly performed studies to determine blood glucose meter accuracy/performance are needed rather than extensive inappropriately designed internal local validation studies.
